# Artificial intelligence-assisted remote detection of ST-elevation myocardial infarction using a mini-12-lead electrocardiogram device in prehospital ambulance care

**DOI:** 10.3389/fcvm.2022.1001982

**Published:** 2022-10-14

**Authors:** Ke-Wei Chen, Yu-Chen Wang, Meng-Hsuan Liu, Being-Yuah Tsai, Mei-Yao Wu, Po-Hsin Hsieh, Jung-Ting Wei, Edward S. C. Shih, Yi-Tzone Shiao, Ming-Jing Hwang, Ya-Lun Wu, Kai-Cheng Hsu, Kuan-Cheng Chang

**Affiliations:** ^1^Division of Cardiovascular Medicine, Department of Medicine, China Medical University Hospital, Taichung, Taiwan; ^2^Graduate Institute of Biomedical Sciences, China Medical University, Taichung, Taiwan; ^3^Division of Cardiovascular Medicine, Asia University Hospital, Taichung, Taiwan; ^4^Department of Medical Laboratory Science and Biotechnology, Asia University, Taichung, Taiwan; ^5^AI Center for Medical Diagnosis, China Medical University Hospital, Taichung, Taiwan; ^6^School of Post-Baccalaureate Chinese Medicine, China Medical University, Taichung, Taiwan; ^7^Department of Chinese Medicine, China Medical University Hospital, Taichung, Taiwan; ^8^Ever Fortune AI Co., Ltd., Taichung, Taiwan; ^9^School of Medicine, China Medical University, Taichung, Taiwan; ^10^Institute of Biomedical Sciences, Academia Sinica, Taipei, Taiwan; ^11^Center of Institutional Research and Development, Asia University, Taichung, Taiwan

**Keywords:** artificial intelligence (AI), contact-to-balloon (C2B) time, convolutional neural network and long short-term memory (CNN-LSTM), prehospital 12-lead ECGs, ST-elevation myocardial infarction (STEMI)

## Abstract

**Objective:**

To implement an all-day online artificial intelligence (AI)-assisted detection of ST-elevation myocardial infarction (STEMI) by prehospital 12-lead electrocardiograms (ECGs) to facilitate patient triage for timely reperfusion therapy.

**Methods:**

The proposed AI model combines a convolutional neural network and long short-term memory (CNN-LSTM) to predict STEMI on prehospital 12-lead ECGs obtained from mini-12-lead ECG devices equipped in ambulance vehicles in Central Taiwan. Emergency medical technicians (EMTs) from the 14 AI-implemented fire stations performed the on-site 12-lead ECG examinations using the mini portable device. The 12-lead ECG signals were transmitted to the AI center of China Medical University Hospital to classify the recordings as “STEMI” or “Not STEMI”. In 11 non-AI fire stations, the ECG data were transmitted to a secure network and read by available on-line emergency physicians. The response time was defined as the time interval between the ECG transmission and ECG interpretation feedback.

**Results:**

Between July 17, 2021, and March 26, 2022, the AI model classified 362 prehospital 12-lead ECGs obtained from 275 consecutive patients who had called the 119 dispatch centers of fire stations in Central Taiwan for symptoms of chest pain or shortness of breath. The AI's response time to the EMTs in ambulance vehicles was 37.2 ± 11.3 s, which was shorter than the online physicians' response time from 11 other fire stations with no AI implementation (113.2 ± 369.4 s, *P* < 0.001) after analyzing another set of 335 prehospital 12-lead ECGs. The evaluation metrics including accuracy, precision, specificity, recall, area under the receiver operating characteristic curve, and F1 score to assess the overall AI performance in the remote detection of STEMI were 0.992, 0.889, 0.994, 0.941, 0.997, and 0.914, respectively. During the study period, the AI model promptly identified 10 STEMI patients who underwent primary percutaneous coronary intervention (PPCI) with a median contact-to-door time of 18.5 (IQR: 16–20.8) minutes.

**Conclusion:**

Implementation of an all-day real-time AI-assisted remote detection of STEMI on prehospital 12-lead ECGs in the field is feasible with a high diagnostic accuracy rate. This approach may help minimize preventable delays in contact-to-treatment times for STEMI patients who require PPCI.

## Introduction

Acute ST-segment elevation myocardial infarction (STEMI) remains a tremendous global health issue requiring early diagnosis and timely reperfusion for morbidity and mortality reduction. Adherence to guideline-directed strategies, such as performance of prehospital 12-lead electrocardiograms (ECGs), shortening of contact-to-balloon and door-to-balloon times, repetitive monitoring and feedback of predefined quality indicators, and the use of standardized medications, are crucial factors for improving outcomes in patients with STEMI (1, 2). Among these factors, the performance and transmission of prehospital ECGs can expedite patient triage and early recognition of STEMI, thereby contributing to the shortening of contact-to-balloon time. Using on-site ECG transmission *via* the emergency medical system (EMS), STEMI patients could be transferred to the nearest interventional center by ambulance, bypassing the emergency department, and undergo timely catheter-based reperfusion therapy upon prehospital STEMI diagnosis. To improve the efficiency and accuracy of prehospital 12-lead ECG diagnosis, new technologies, including machine learning and deep learning algorithms, have been implemented (3–5).

The application of machine learning or deep learning algorithms to predict STEMI has recently gained considerable attention (4, 6, 7). One of the challenges in the development of such models is the lack of robust 12-lead STEMI datasets for training and validation. As such, most prior algorithms (8–19) have used the open-source ECG database (MIT-BIH PhysioNET or PTB Physiobank), or have retrospectively analyzed historical ECGs from hospital records with a relatively small number of verified STEMI 12-lead ECGs, for model development.

Although most of these machine learning classifiers report a high accuracy rate of >90%, whether the usefulness of such models can be generalizable to real-world clinical settings remains questionable. An alternative approach for developing these models is to combine classical ECG features with clinical patient data, such as medical history and laboratory parameters (20–23). Despite the high accuracy achieved by these models, the clinical utility in early prehospital patient triage is often limited owing to the need for clinical data, according to their original algorithm designs. Recently, Al-Zaiti et al. (24) trained and tested multiple classifiers on two independent prospective patient cohorts using prehospital 12-lead ECGs, which outperformed experienced clinicians and the interpretations made by commercial software. However, their results were based on offline analysis, and they have yet to be confirmed in real-world online practice.

We previously utilized a bidirectional long short-term memory deep learning model to detect STEMI and 12 major heart rhythms, which outperformed board-certified physicians including cardiologists, emergency physicians, and internists (4, 6). We further developed a new model combining a convolutional neural network and long short-term memory (CNN-LSTM), and implemented an all-day artificial intelligence (AI)-based triage system to facilitate STEMI ECG diagnosis in the ED (5). We implemented the CNN-LSTM model to detect STEMI on prehospital 12-lead ECGs acquired from a mini portable ECG device in an ambulance service to expedite patient triage and support decision-making. The purpose of this study was to provide online real-world evidence by assessing how AI-assisted remote detection of STEMI may impact the timeliness and accuracy of prehospital ECG interpretation, important elements related to early diagnosis and timely reperfusion in STEMI patients.

## Methods

### Prehospital 12-lead ECG workflow

**Figure 1:**
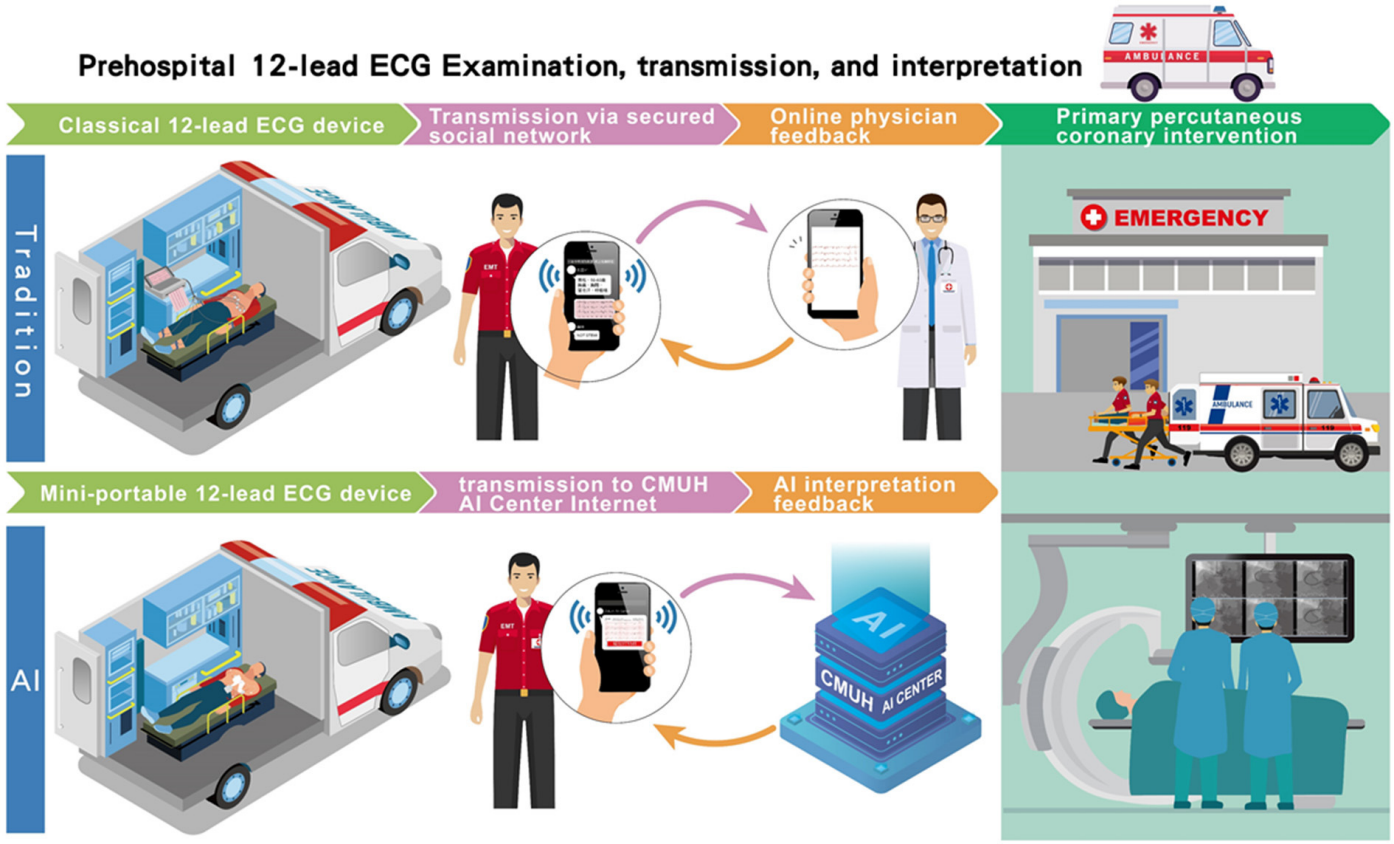
The flowchart of the AI-based pre-hospital STEMI detection system. Traditionally, after the 12-lead ECG had been recorded in the ambulance vehicle, the ECG data were posted on a secured network for reading by available online physicians as had been usual practice. The time interval between ECG transmission and interpretation feedback by physicians was defined as the physician's response time. In our AI-based pre-hospital STEMI detection system, the recorded signal was also simultaneously transmitted to the AI center of the China Medical University Hospital to be classified “STEMI” or “Not STEMI.” Similarly, the time interval between the ECG transmission and the ECG interpretation feedback by the AI was defined as the AI's response time.

The implementation of AI-based STEMI detection on prehospital 12-leads ECG was conducted in Taichung City and Nantou County in Central Taiwan between July 17, 2021, and March 26, 2022. A total of 14 pre-assigned fire stations, covering 19% of all fire stations and serving an estimated population size of 562,222, in the two administrative districts participated this pilot study. During the study period, we analyzed prehospital 12-lead ECG data recorded in ambulance vehicles from patients who called 119 dispatch centers of fire stations for symptoms of chest pain or shortness of breath in Taichung City and Nantou County. Emergency medical technicians (EMTs) from the 14 pre-assigned fire stations performed the on-site 12-lead ECG examinations using mini portable devices (QT Medical, Diamond Bar, CA., USA), after pre-on-board training. Individuals who had the characteristics including traumatic chest or abdominal injury, conscious disturbance, failure to cooperate with or refusal of ECG examinations, age ≤18 year-old, and pregnant women were excluded from the ECG examination. This portable ECG machine is a U.S. Food and Drug Administration-cleared and CE marking device that can be used for personal care and provide hospital-grade 12-lead electrocardiograms. In contrast to the conventional 12-lead ECG machine, which requires placing ten separate electrodes and connecting 10 lead wires, this mini portable ECG device uses a single-piece disposable electrode design with no lead wires to provide certified digital 12-lead ECG signals. After the 12-lead ECG had been recorded in the ambulance vehicle, the ECG signal was first transmitted to the AI center of the China Medical University Hospital to be classified “STEMI” or “Not STEMI.” The AI-annotated ECG data were then posted on a secured network for diagnosis confirmation by available online emergency physicians as had been usual practice. The time interval between the ECG transmission and the ECG interpretation feedback by the AI was defined as the AI's response time (Figure 1). Similarly, the time interval between ECG transmission and interpretation feedback by physicians was defined as the physician's response time. The physicians' response times at 11 other fire stations without AI implementation were collected for comparison. When the AI model identified STEMI on a prehospital ECG and the result was confirmed by available online physicians, the EMT personnel contacted the nearest available interventional hospital to shorten ambulance transfer time for timely reperfusion therapy.

### AI model for detection of STEMI on prehospital 12-lead ECGs

The development of our deep learning model to classify STEMI on 12-lead ECGs has been previously reported (4, 5). Briefly, we first retrieved 3,296 12-lead ECG data from the digital ECG core laboratory database at the China Medical University Hospital (CMUH) in an extensible markup language (XML) format as inputs to develop the AI model. The 12-lead ECGs were recorded at a sampling rate of 500 Hz using a computerized ECG machine (GE Healthcare MAC 2000/3500/5500, US). After excluding 389 ECGs with significant noise or artifacts, the remaining 2,907 ECGs containing 882 STEMI ECGs and 2,025 non-STEMI ECGs, as judged by the ground truth committee, were used for model training (80%) and validation (20%).

**Figure 2:**
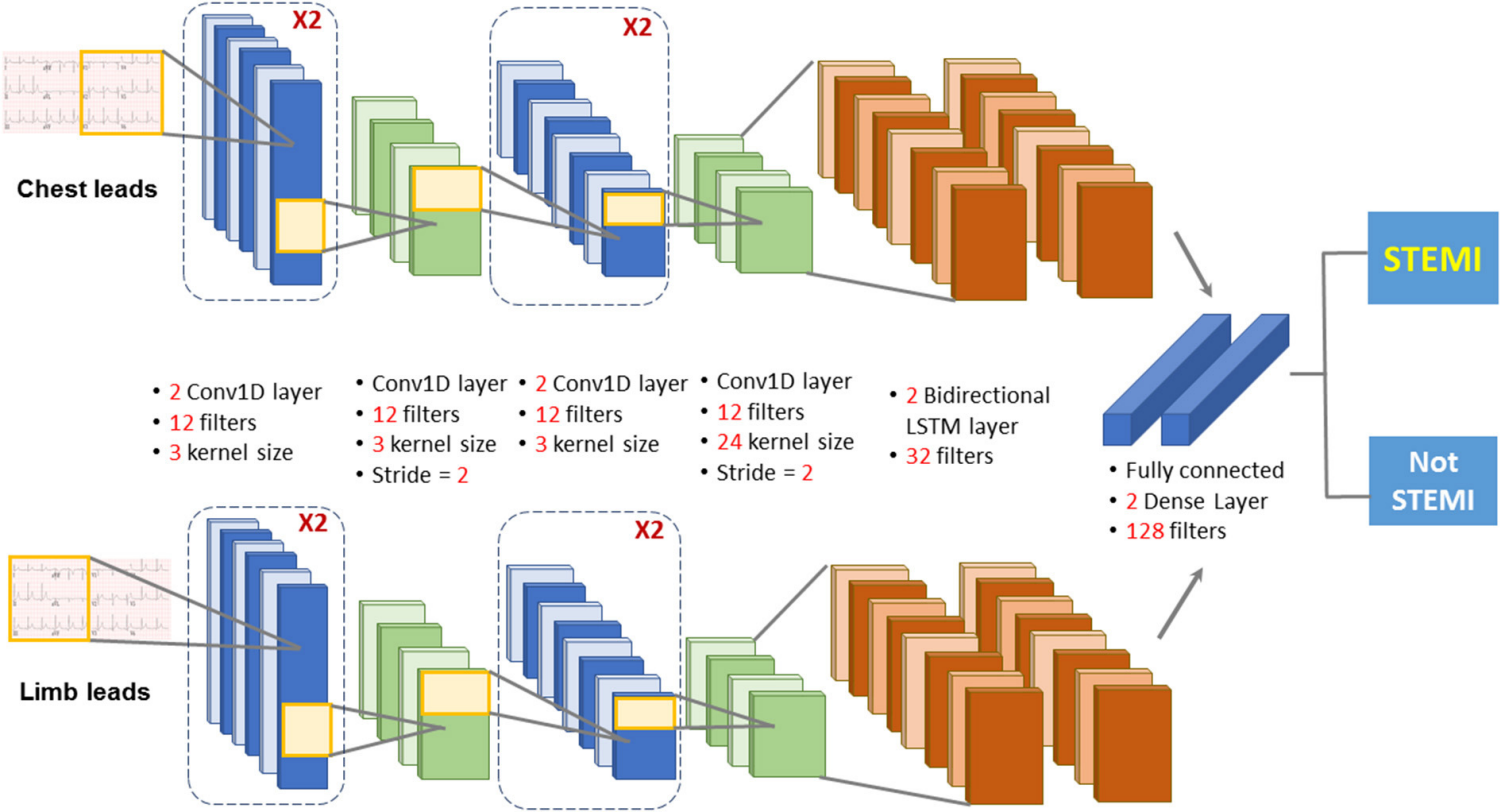
Diagram depicting the deep learning model architecture. The deep learning model used a combination of CNN and LSTM to classify STEMI on 12-lead ECGs. The architecture of the proposed AI mode was composed of two 1D -CNNs blocks fed with chest and limb leads, to extract the features from the 6-lead signals. The outputs of the two 1D-CNNs were connected to two layers of LSTM, which served as a sequence analyzer. Then the outputs of the two LSTMs were concatenated and connected to a fully connected layer to classify the data as “STEMI” or “Not STEMI”.

The deep learning model used a combination of CNN and LSTM to classify STEMI on 12-lead ECGs. The architecture of the proposed AI mode (CNN-LSTM) described previously (25) was composed of two 1D-CNNs blocks fed with chest and limb leads, to extract the features from the 6-lead signals (Figure 2). The outputs of the two 1D-CNNs were connected to two layers of LSTM, which served as a sequence analyzer. Then the outputs of the two LSTMs were concatenated and connected to a fully connected layer to classify the data as “STEMI” or “Not STEMI.” In the training module, we chose the binary cross-entropy as the loss function, and the Adam as the optimizer. By combining the strengths of CNN and LSTM architectures, the current CNN-LSTM model was developed to predict STEMI on the prehospital 12-lead ECG because of its high spatio-temporal dependencies. The precision, F1 scores, and recall were evaluated using the validation set to prevent overfitting during the training process. Before deploying the STEMI classifier model, an additional 4,007 12-lead ECGs were tested against the ground truth with excellent overall performance, as previously reported (5). The overall performance of the AI model in classifying “STEMI” or “Not STEMI” on all prehospital 12-lead ECGs was assessed by a confusion matrix, and the accuracy, precision, recall, area under the receiver operating characteristic (ROC) curve, and F1 score against a ground truth were assessed according to the consensus of three expert board-certified cardiologists.

### Validation of 12-lead ECG signals between devices

**Figure 3:**
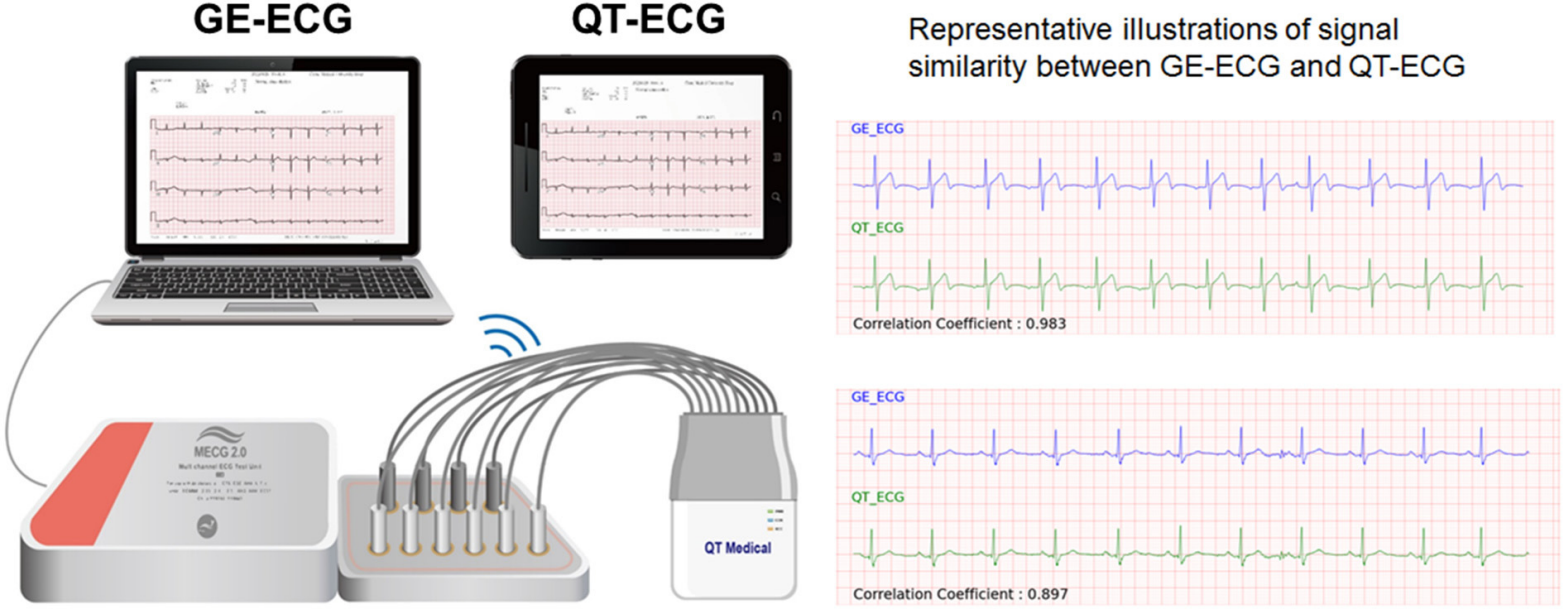
Validation of 12-lead ECG signals between devices. The proposed deep learning model for STEMI detection was based on the digital 12-lead ECG signals recorded using a computerized ECG machine (GE Healthcare MAC 2000/3500/5500, USA). For the prehospital 12-lead ECG acquisition in the current study, we used a mini portable ECG device (QT Medical, Diamond Bar, CA, USA) with the proposed AI algorithm integrated within. To ensure the efficacy of AI-based STEMI detection using the mini-portable ECG device, we checked the consistency of the 12-lead ECG output signals between the two devices. A total of 194 verified ECGs acquired from GE machines (GE-ECGs) were converted into the corresponding QT Medical ECG output format (QT-ECGs) using a certified ECG simulator. Finally, the signal similarities between raw GE-ECGs and the transcribed QT-ECGs was analyzed. The performance of the AI model in classifying data as “STEMI” or “Not STEMI” for each of the two sets of ECG signals was compared to attest to the consistency of AI performance across devices.

The proposed deep learning model for STEMI detection was based on the digital 12-lead ECG signals recorded using a computerized ECG machine (GE Healthcare MAC 2000/3500/5500, US). For the prehospital 12-lead ECG acquisition in the current study, we used a mini portable ECG device (QT Medical, Diamond Bar, CA., USA) with the proposed AI algorithm integrated within. To ensure the efficacy of AI-based STEMI detection using the mini-portable ECG device, we checked the consistency of the 12-lead ECG output signals between the two devices. We randomly retrieved a separate set of 199 12-lead ECGs including 99 “STEMI” and 100 “Not-STEMI” data from the digital ECG core laboratory database of CMUH. After excluding five ECGs with excessive noise, each of the remaining 194 ECGs acquired from GE machines (GE-ECGs) were converted into the corresponding QT Medical ECG output format (QT-ECGs) using a certified ECG simulator (WhaleTeq Co., Ltd, Taipei, Taiwan) (Figure 3). Finally, the signal similarities between raw GE-ECGs and the transcribed QT-ECGs was analyzed. The performance of the AI model in classifying data as “STEMI” or “Not STEMI” for each of the two sets of ECG signals was compared to attest to the consistency of AI performance across devices.

### Statistical analysis

Continuous data with normal distribution are expressed as the mean ± standard deviation, while non-normally distributed data are reported as median (25th−75th percentiles). Differences in data were analyzed using the Student's t-test or the Mann-Whitney U test as appropriate. Categorical data were expressed as numbers (percentages) and were compared using the chi-square test or Fisher's exact test. A two-tailed probability value of <0.05 was considered statistically significant. The correlation coefficient was used to assess the signal similarity between GE-ECGs and QT-ECGs. This score ranged from 1 and −1, where 1 indicated a perfect positive correlation, −1 represented a perfect negative correlation, and 0.7–1 denoted a highly positive correlation. The Cohen Kappa value was also used to verify the consistency of the AI's performance between the raw GE-ECG and transcribed QT-ECG signals. This value ranged from 1 to −1, and scores between 0.81 and 1.00 indicated an almost perfect agreement. All statistical analyses were performed using the SAS software (version 9.4; SAS Institute, Cary, NC, USA). The Research Ethics Committee of CMUH reviewed and approved the study protocol (IRB: CMUH111-REC2-104).

## Results

**Figure 4:**
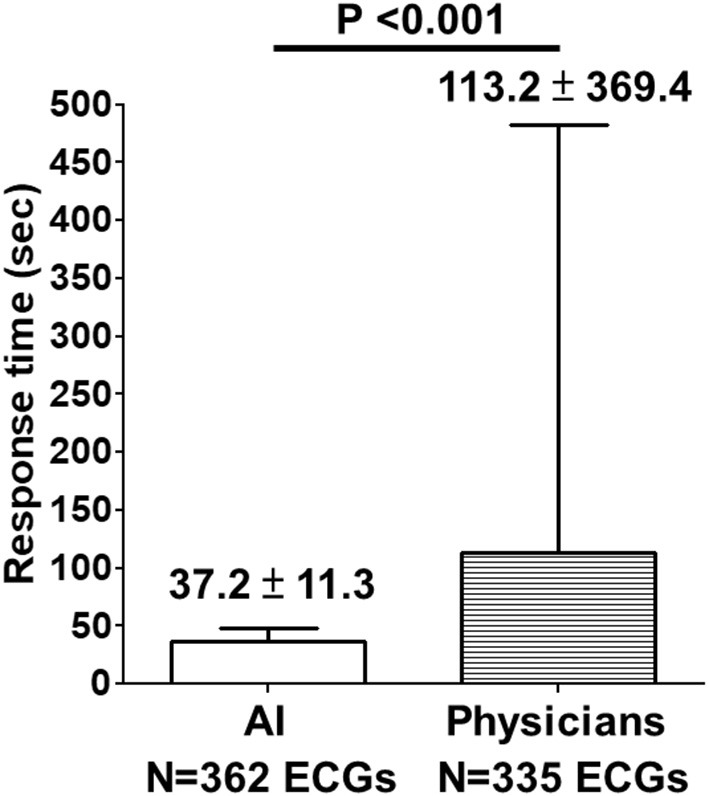
Comparison of response time between AI and physicians on prehospital ECGs. Between July 17, 2021, and March 26, 2022, the proposed AI model classified a total of 362 prehospital 12-lead ECGs as “STEMI” or “Not STEMI”, obtained from 275 consecutive patients who had called the 119 dispatch centers of the fire stations in Taichung City and Nantou County for symptoms of chest pain or shortness of breath. The AI's response time to the EMTs in ambulance vehicles was 37.2 ± 11.3 s, which was shorter than the online physicians' response time (113.2 ± 369.4 s, *P* < 0.001) from 11 other fire stations with no AI implementation after analyzing another set of 335 prehospital 12-lead ECGs.

Between July 17, 2021, and March 26, 2022, the proposed AI model classified a total of 362 prehospital 12-lead ECGs as “STEMI” or “Not STEMI,” obtained from 275 consecutive patients who had called the 119 dispatch centers of the fire stations in Taichung City and Nantou County for symptoms of chest pain or shortness of breath. The AI's response time to the EMTs in ambulance vehicles was 37.2 ± 11.3 s, which was shorter than the online physicians' response time from 11 other fire stations with no AI implementation (113.2 ± 369.4 s, *P* < 0.001) after analyzing another set of 335 prehospital 12-lead ECGs (Figure 4).

**Figure 5:**
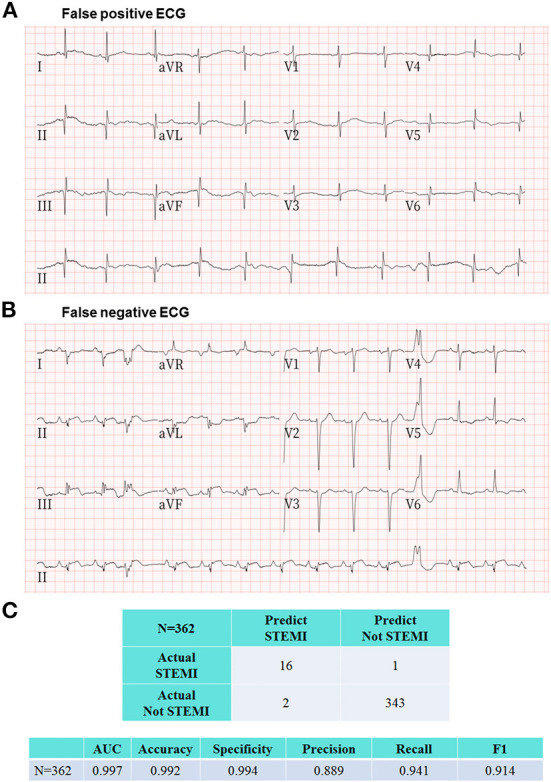
Representative ECGs of false positive and false negative labeling by the proposed AI model. **(A)** This prehospital ECG showing pathological Q waves in the inferior and anterolateral leads was classified as STEMI by AI. The ground truth committee interpreted this ECG as recent or old myocardial infarctions and judged this AI labeling as a false positive case. **(B)** There was only one ECG with a false negative labeling by AI as “Not STEMI,” which was interpreted as “STEMI” according to the adjudication by the ground truth committee. Interestingly, this patient was diagnosed with a recent myocardial infarction by the cardiologist in charge at the destination hospital after incorporating more hospital-based information including historical ECGs and laboratory data, and did not require PPCI. **(C)** The evaluation metrics including area under the receiver operating characteristic curve, accuracy, specificity, precision, recall, and F1 score to assess the overall AI performance in the remote detection of STEMI from 362 prehospital 12-lead ECGs were 0.997, 0.992, 0.994, 0.889, 0.941, and 0.914, respectively.

Among the 362 prehospital 12-lead ECGs, AI labeled 18 as “STEMI,” and the remaining 344 ECGs as “Not STEMI.” Of the 18 AI STEMI labeled ECGs, 16 were interpreted as STEMI, and the remaining two were judged as false-positives according to a consensus of board-certified cardiologists. Among the 12 adjudicated STEMI patients, 9 patients were diagnosed based on a single ECG, and the remaining 3 patients had received multiple ECGs (3 ECGs in one, and 2 ECGs in another 2 patients) with all ECGs showing consistent STEMI features. Ultimately, 10 out of the 12 adjudicated STEMI patients underwent primary percutaneous coronary intervention (PPCI) with a median contact-to-door time of 18.5 (IQR: 16–20.8) mins and a median contact-to-balloon time of 92.5 (IQR: 81–124.8) mins (Table 1). Of the remaining two correctly labeled STEMI patients, one was confirmed by coronary angiography to have acute myocarditis-related ST elevations without coronary artery occlusion, and the other was judged to be a recent myocardial infarction based on the comparison of serial ECGs and laboratory data, and did not receive PPCI at the discretion of the cardiologist in charge at the destination hospital. Both ECGs with a false positive STEMI classification by AI were judged to be recent or old myocardial infarctions by the ground truth committee (Figure 5). One ECG with a false negative labeling by AI as “Not STEMI,” was interpreted as “STEMI” according to the adjudication by the ground truth committee (Figure 5). Interestingly, this patient was diagnosed with a recent myocardial infarction by the cardiologist in charge at the destination hospital after incorporating more hospital-based information, such as historical ECGs for comparison and laboratory data, and did not require PPCI. The evaluation metrics including accuracy, precision, specificity, recall, area under the receiver operating characteristic curve, and F1 score to assess the overall AI performance in the remote detection of STEMI from 362 prehospital 12-lead ECGs were 0.992, 0.889, 0.994, 0.941, 0.997, and 0.914, respectively (Figure 5).

**Table 1:** Consistent labeling of ST-elevation myocardial infarction by AI and ground truth committee (12 cases).

**Patient No**.	**Demographics**		**Quality metrics**					**Outcomes**	
	**Age**	**Gender**	**AI STEMI**	**Consensus STEMI**	**Contact-to-ECG (min)**	**Contact-to-door (min)**	**Contact-to-balloon (min)**	**Culprit vessel revascularization**	**Hospital discharge**
1	53	M	Y	Y	10	21	83	Proximal RCA	Alive
2	55	M	Y	Y	8	19	143	LM to LAD; Cardiogenic shock with ECMO	Expired
3	68	M	Y	Y	14	20	131	Proximal LCX	Alive
4	67	F	Y	Y	5	14	81	Middle LAD	Alive
5	89	M	Y	Y	5	10	82	Middle LAD	Alive
6	63	M	Y	Y	6	17	69	Proximal RCA; VT/VF with DC shock and IABP	Alive
7	45	M	Y	Y	10	21	74	Middle RCA	Alive
8	53	M	Y	Y	8	18	106	Middle RCA	Alive
9	76	M	Y	Y	11	23	102	Proximal RCA	Alive
10	58	M	Y	Y	9	16	275	LM to LAD; OHCA with ECMO	Expired
11	65	F	Y	Y	12	16	-	Myocarditis with cardiogenic shock and ECMO	Expired
12	62	M	Y	Y	11	22	-	Old MI, No PPCI	Alive

Contact-to-ECG time, time between emergency medical technicians (EMT) arrival and first ECG performed; Contact-to-Door time, time between EMT arrival and patient arrival at the hospital; Contact-to-Balloon time, time between EMT arrival and restoration of coronary artery blood flow; RCA, right coronary artery; LM, left main coronary artery; LAD, left anterior descending artery; ECMO, extra-corporeal membrane oxygenation; LCX, left circumflex artery; VT/VF, ventricular tachycardia/ventricular fibrillation; DC, direct current; IABP, intra-aortic balloon pump; OHCA, out-of-hospital cardiac arrest; MI, myocardial infarction; PPCI, primary percutaneous coronary intervention.

Before applying the AI algorithm, which was originally developed using GE-ECG data to predict the prehospital 12-lead ECG signals acquired from the QT-ECG device in ambulance vehicles, we first checked the signal similarity and consistency of AI performance between the two devices used. Among the separate set of 194 12-lead ECGs with 2,328 ECG leads signals, the correlation coefficient of output signals from GE-ECGs and QT-ECGs was >0.85 in 94.2% (2,193) ECGs, indicating that they were highly correlated. In addition, the Cohen Kappa value for testing the consistency of AI performance between the sets of ECG signals was 0.88, representing an almost perfect agreement (Figure 3).

## Discussion

To the best of our knowledge, the current study is the first real-world study to implement a 24/7 AI-based algorithm to detect STEMIs on prehospital 12-lead ECGs to facilitate patient triage and ensure timely reperfusion therapy by shortening the contact-to-door. The strengths of this study, in contrast to conventional strategies, are that the proposed AI model provides a cardiologist-level STEMI ECG diagnosis using a simple-to-use mini ECG device that does not require additional manpower for expediting patient triage in a real-world prehospital setting.

### The importance of prehospital 12-lead ECG

STEMI is a medical emergency requiring early diagnosis and timely reperfusion treatment by expert teams in experienced centers. The performance of prehospital 12-lead ECGs is a key element in enabling early diagnosis and transfer of STEMI patients to a PPCI-capable hospital. On the other hand, if STEMI patients are inappropriately transferred to a PPCI-incapable hospital, this can cause a significant delay in time to reperfusion, ~60–80 mins (26–31). Once STEMI was identified by prehospital ECGs, early notification of the emergency department resulted in a significant shortening of the first medical contact-to-reperfusion time, door-to-balloon time, and door-to-needle time compared with no prehospital 12-lead ECG recording in STEMI patients (32–42). The results of our study concur with these findings, with a fast contact-to-door time and contact-to-balloon time in 10 identified STEMI patients undergoing PPCI compared with registry data from the USA and Europe. The shortening of contact-to-reperfusion time was even more striking when excluding the two patients presenting with cardiac arrest, with one occurring in the field and the other in the emergency department. Furthermore, with early detection of STEMI on prehospital ECGs, STEMI patients can be directly transferred to the cardiac catheterization room, bypassing the emergency department for PPCI to achieve prompt reperfusion treatment (37). Therefore, the contemporary American College of Cardiology/American Heart Association and European Society of Cardiology guidelines provide Class I recommendations for performing prehospital 12-lead ECGs and notifying the emergency care service in advance (43, 44).

### AI-based autodetection of STEMI on prehospital ECG

Despite the recognition of the importance and the increasing utilization of the prehospital 12-lead ECG in the field, performing and accurately interpreting prehospital ECGs remains challenging. A 12-lead ECG examination in a prehospital setting is almost exclusively performed by EMT personnel. With the conventional ECG device, it usually takes time and training for paramedics to place 10 separate electrodes and connect 10 lead wires correctly, which may limit the widespread utilization of prehospital ECG for field triage (45). In the current study, we were the first to use a mini-ECG device with a single-piece disposable electrode design and proven certified digital 12-lead ECG signals. Its easy-to-use design greatly motivated the paramedics to perform prehospital 12-lead ECG, according to a user satisfaction survey conducted during the study period (data not shown), along with a relatively short first EMT contact-to-ECG time.

In the USA and Europe, interpretation of the prehospital 12-lead ECG was usually made by the device's computerized algorithm or initially by trained paramedics, and then the ECGs were transmitted wirelessly *via* the Internet to the PPCI center for confirmation. Recently, machine-learning-based algorithms have been proposed to assist in the prehospital diagnosis of acute coronary syndrome (ACS), including STEMI. Takeda et al. employed a machine learning-based approach for early prediction of ACS using 17 features, including vital signs, 3-lead ECG monitoring, and symptoms, and demonstrated that this model was highly predictive of ACS in a prehospital setting. However, they built a predictive algorithm using a 3-lead, and not a 12-lead ECG, and whether the model improved the timeliness of ACS diagnosis was not presented in this study (46). Al-Zaiti et al. utilized features extracted only from the prehospital 12-lead ECGs, achieving a machine learning model that outperformed both commercial software and experienced clinicians in the prehospital diagnosis of ACS. Nevertheless, the low positive predictive rate (0.43) of this model and the additional requirement of combined judgment from trained EMT personnel may limit its clinical utility (24).

The current study has several strengths in bridging the existing gaps to fulfill the guideline-recommended performance and timely interpretation of prehospital 12-lead ECG to expedite coronary reperfusion. First, we integrated a useful AI algorithm into an easy-to-use 12-lead ECG device that motivates EMT personnel to perform prehospital 12-lead ECG examinations and may further shorten contact-to-ECG time. Second, in this study, although the mean differences of the response time between AI and physicians was <2 min, we noticed high variations of the physician's response times with some of the physician's response times were far longer than the mean value. While the AI response time showed little variations, 5% of the physician's response time was >10 min in fire stations with no AI implementation. This finding highlights a potential limitation of physician-dependent reading of the prehospital ECGs. Third, the performance of our AI model, which reached a cardiologist level of STEMI detection, has been extensively validated in preclinical testing and in emergency medicine environments. Implementation of the AI algorithm can facilitate chest pain triage, shorten the D2B time during off-hours, and increase the percentage of D2B time <90 min in the emergency department. Forth, before implementing the AI model in the mini portable ECG device, we checked and confirmed the signal similarity and consistency of the AI performance between the GE-ECG and QT-ECG devices, which is essential for ensuring the uniformity of AI performance in the field. Taken together, this pilot study is the first to report an AI-assisted diagnosis of STEMI on prehospital 12-lead ECGs and its potential impact on time-to-treatment for patients with STEMI.

### Limitations

Our study had several limitations. First, the proposed AI algorithm was developed to interpret STEMI in a binary response model. Thus, it is currently not possible for this model to detect other types of ACS beyond STEMI, such as non-STEMI or unstable angina. However, a large body of evidence has shown that STEMI remains the most time-critical condition, requiring prompt intervention for revascularization, compared with other types of ACS (1, 2). Therefore, the proposed AI model was intended solely for assisting with STEMI triage in consideration of its clinical importance in a prehospital setting. Second, although the AI-based approach seems to have a short prehospital transport time, such as contact-to-ECG, contact-to-door, or contact-to-balloon times, whether these results can be translated into better outcomes needs to be confirmed in large-scale controlled studies. Third, the study was conducted in two administrative areas in Central Taiwan, with a limited number of fire stations participating in the program. The usefulness of this AI-based approach in assisting with the prehospital diagnosis of STEMI needs to be confirmed in a prospective cohort involving a broader geographic region or across entire countries. Finally, only 10 STEMI patients identified by this program underwent PPCI, and it is still too early to draw a solid conclusion that the AI-based remote clinical decision support system may contribute to better outcomes in STEMI patients. Nevertheless, the results of the current study demonstrated the feasibility and excellent performance of the proposed AI model in classifying STEMI on prehospital 12-lead ECGs.

## Conclusions

We demonstrated the feasibility and usefulness of implementing AI-assisted remote STEMI detection with prehospital 12-lead ECG using a mini portable ECG device in the field. This strategy can facilitate the prehospital STEMI diagnosis, and may help minimize preventable delays in contact-to-treatment time for patients with STEMI undergoing PPCI.

## Data availability statement

The original contributions presented in the study are included in the article/supplementary material, further inquiries can be directed to the corresponding author/s.

## Ethics statement

The studies involving human participants were reviewed and approved by Research Ethics Committee of China Medical University Hospital (IRB: CMUH111-REC2-104). Written informed consent for participation was not required for this study in accordance with the national legislation and the institutional requirements.

## Author contributions

K-WC and Y-CW: conception and design, provision of study material or patients, data analysis and interpretation, and manuscript writing. B-YT, M-HL, Y-LW, and K-CH: collection of data and data analysis and interpretation. M-YW, ES, M-JH, and Y-TS: data analysis and interpretation. K-CC: financial support, conception and design, provision of study material or patients, data analysis and interpretation, and manuscript writing. All authors contributed to the article and approved the submitted version.

## Funding

This study was supported in part by the Taiwan Ministry of Science and Technology (MOST 111-2314-B-039-012, MOST 110-2314-B-039-050, and MOST 109-2314-B-039-045), China Medical University Hospital (DMR-108-013, DMR-109-012, and DMR-110-012), and Asia University Hospital (10851014, 10951005, and 11051001). None of these funding sources played any role in the study design, collection, analysis, interpretation of the data, writing of the report, or in the decision to submit the paper for publication.

## Conflict of interest

Author P-HH was employed by Ever Fortune AI Co., Ltd.

The remaining authors declare that the research was conducted in the absence of any commercial or financial relationships that could be construed as a potential conflict of interest.

## Publisher's note

All claims expressed in this article are solely those of the authors and do not necessarily represent those of their affiliated organizations, or those of the publisher, the editors and the reviewers. Any product that may be evaluated in this article, or claim that may be made by its manufacturer, is not guaranteed or endorsed by the publisher.
