# Corrigendum: Artificial intelligence-assisted remote detection of ST-elevation myocardial infarction using a mini-12-lead electrocardiogram device in prehospital ambulance care

**DOI:** 10.3389/fcvm.2022.1078223

**Published:** 2022-12-09

**Authors:** Ke-Wei Chen, Yu-Chen Wang, Meng-Hsuan Liu, Being-Yuah Tsai, Mei-Yao Wu, Po-Hsin Hsieh, Jung-Ting Wei, Edward S. C. Shih, Yi-Tzone Shiao, Ming-Jing Hwang, Ya-Lun Wu, Kai-Cheng Hsu, Kuan-Cheng Chang

**Affiliations:** ^1^Division of Cardiovascular Medicine, Department of Medicine, China Medical University Hospital, Taichung, Taiwan; ^2^Graduate Institute of Biomedical Sciences, China Medical University, Taichung, Taiwan; ^3^Division of Cardiovascular Medicine, Asia University Hospital, Taichung, Taiwan; ^4^Department of Medical Laboratory Science and Biotechnology, Asia University, Taichung, Taiwan; ^5^AI Center for Medical Diagnosis, China Medical University Hospital, Taichung, Taiwan; ^6^School of Post-Baccalaureate Chinese Medicine, China Medical University, Taichung, Taiwan; ^7^Department of Chinese Medicine, China Medical University Hospital, Taichung, Taiwan; ^8^Ever Fortune AI Co., Ltd., Taichung, Taiwan; ^9^School of Medicine, China Medical University, Taichung, Taiwan; ^10^Institute of Biomedical Sciences, Academia Sinica, Taipei, Taiwan; ^11^Center of Institutional Research and Development, Asia University, Taichung, Taiwan

**Keywords:** artificial intelligence (AI), contact-to-balloon (C2B) time, convolutional neural network and long short-term memory (CNN-LSTM), prehospital 12-lead ECGs, ST-elevation myocardial infarction (STEMI)

In the original article, there was an error in Figure 1: “The flowchart of the AI-based pre-hospital STEMI detection system” as published. A typo error in the figure read “Prehospital 12-lead ECG examinectin,” and has been corrected to “Prehospital 12-lead ECG examination.” The corrected figure appears below.

**Figure 1:**
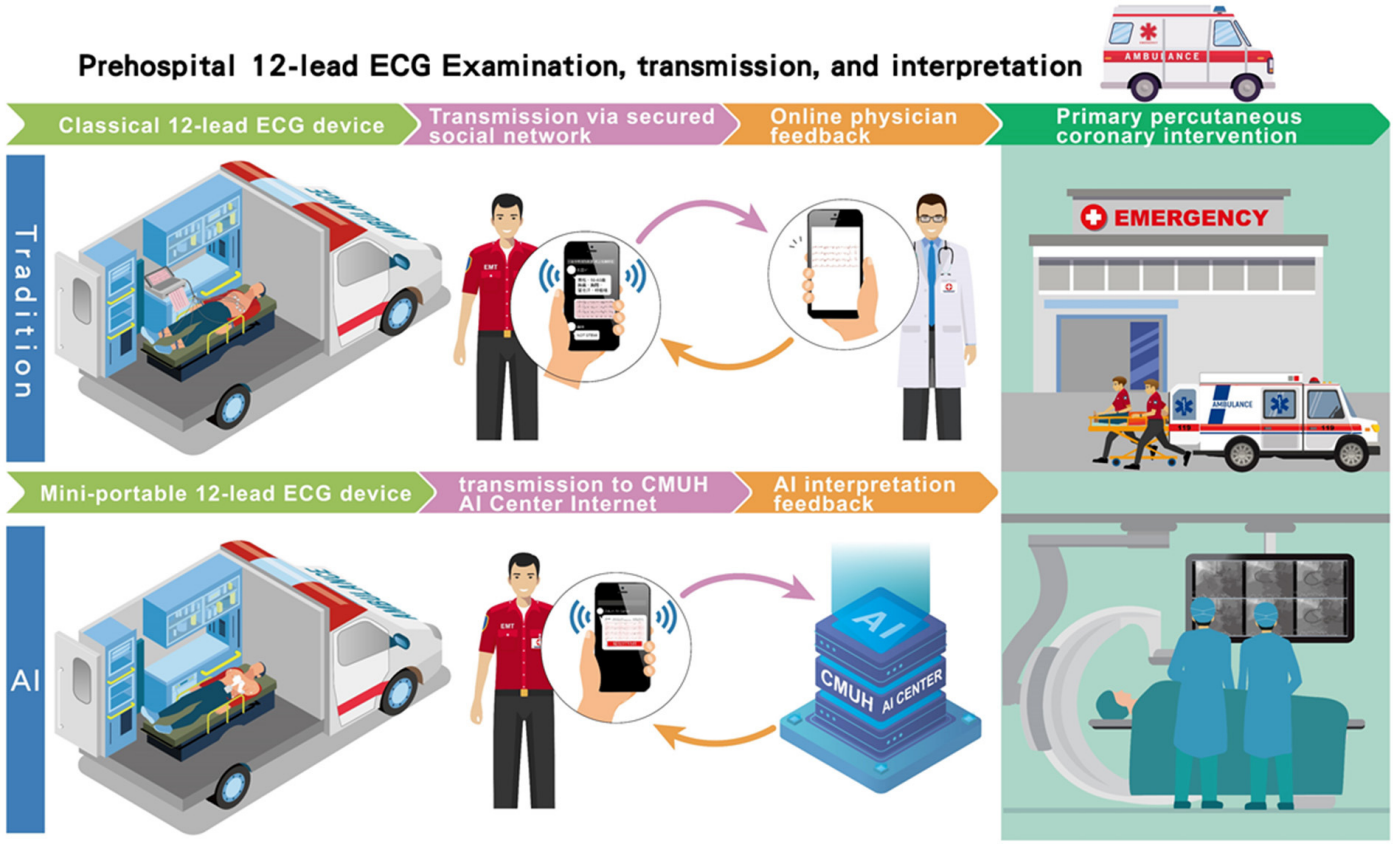
The flowchart of the AI-based pre-hospital STEMI detection system. Traditionally, after the 12-lead ECG had been recorded in the ambulance vehicle, the ECG data were posted on a secured network for reading by available online physicians as had been usual practice. The time interval between ECG transmission and interpretation feedback by physicians was defined as the physician's response time. In our AI-based pre-hospital STEMI detection system, the recorded signal was also simultaneously transmitted to the AI center of the China Medical University Hospital to be classified “STEMI” or “Not STEMI.” Similarly, the time interval between the ECG transmission and the ECG interpretation feedback by the AI was defined as the AI's response time.

The authors apologize for this error and state that this does not change the scientific conclusions of the article in any way. The original article has been updated.

